# Immune receptor repertoires in pediatric and adult acute myeloid leukemia

**DOI:** 10.1186/s13073-019-0681-3

**Published:** 2019-11-26

**Authors:** Jian Zhang, Xihao Hu, Jin Wang, Avinash Das Sahu, David Cohen, Li Song, Zhangyi Ouyang, Jingyu Fan, Binbin Wang, Jingxin Fu, Shengqing Gu, Moshe Sade-Feldman, Nir Hacohen, Wuju Li, Xiaomin Ying, Bo Li, X. Shirley Liu

**Affiliations:** 1 Center for Computational Biology, Beijing Institute of Basic Medical Sciences, Beijing, China; 2 Department of Data Sciences, Dana-Farber Cancer Institute and Harvard T.H. Chan School of Public Health, Boston, MA USA; 3 Shanghai Key Laboratory of Tuberculosis, Clinical Translational Research Center, Shanghai Pulmonary Hospital, School of Life Sciences and Technology, Tongji University, Shanghai, China; 4 Massachusetts General Hospital Cancer Center, Harvard Medical School (HMS), Boston, MA USA; 5 Broad Institute of Massachusetts Institute of Technology (MIT) and Harvard, Cambridge, MA USA; 6 Department of Medicine, Massachusetts General Hospital, HMS, Boston, MA USA; 7 Lyda Hill Department of Bioinformatics, UT Southwestern Medical Center, Dallas, TX USA

**Keywords:** Acute myeloid leukemia, T cell receptor repertoires, B cell receptor repertoires, Complementarity-determining region 3

## Abstract

**Background:**

Acute myeloid leukemia (AML), caused by the abnormal proliferation of immature myeloid cells in the blood or bone marrow, is one of the most common hematologic malignancies. Currently, the interactions between malignant myeloid cells and the immune microenvironment, especially T cells and B cells, remain poorly characterized.

**Methods:**

In this study, we systematically analyzed the T cell receptor and B cell receptor (TCR and BCR) repertoires from the RNA-seq data of 145 pediatric and 151 adult AML samples as well as 73 non-tumor peripheral blood samples.

**Results:**

We inferred over 225,000 complementarity-determining region 3 (CDR3) sequences in TCR α, β, γ, and δ chains and 1,210,000 CDR3 sequences in B cell immunoglobulin (Ig) heavy and light chains. We found higher clonal expansion of both T cells and B cells in the AML microenvironment and observed many differences between pediatric and adult AML. Most notably, adult AML samples have significantly higher level of B cell activation and more secondary Ig class switch events than pediatric AML or non-tumor samples. Furthermore, adult AML with highly expanded IgA2 B cells, which might represent an immunosuppressive microenvironment, are associated with regulatory T cells and worse overall survival.

**Conclusions:**

Our comprehensive characterization of the AML immune receptor repertoires improved our understanding of T cell and B cell immunity in AML, which may provide insights into immunotherapies in hematological malignancies.

## Background

Acute myeloid leukemia (AML), caused by the abnormal proliferation of immature myeloid cells in the blood or bone marrow (BM), is the most common acute leukemia in adults and the second most common in children [1]. For many years, the standard therapy for AML has been chemotherapy regimens with or without allogeneic hematopoietic stem cell transplantation [2]. This strategy often induces complete remission, but a majority of patients will ultimately relapse and succumb to the disease [2–5]. Advances in immunotherapies, particularly immune checkpoint blockade (ICB) and engineered T cells, have revolutionized cancer therapy in recent years [6, 7]. However, the treatment of AML with immunotherapies so far has been promising but very challenging [8]. In contrast to the success of ICB therapy in many solid tumors, the only published phase I study of pidilizumab (anti-PD1) in AML showed peripheral blast reduction only in one out of eight patients [9]. Though low mutational burden was considered the cause of low endogenous immune responses for ICB treatment in AML [10], the intrinsic resistance mechanisms of the leukemic blasts against immune responses remains poorly understood. In addition, due to the lack of specific target antigen, treatment with chimeric antigen receptor (CAR) T cells is still challenging for AML compared to the prominent effect of CAR T therapies targeting CD19/CD20 in B cell leukemia and lymphoma [11]. Hence, better understanding of the interactions between AML malignant cells and the immune microenvironment has the potential to improve patient outcome and inform novel immunotherapy strategies for AML patients [12].

T cell and B cell are key components of the adaptive immunity. With the development of ICB therapy, the antitumor properties of infiltrating T cells have been well confirmed in many solid tumors such as melanoma and non-small cell lung cancer [6]. Upon binding to tumor neo-antigens, cytotoxic T cells can eliminate the cancer cells [13]. Though infiltrating B cells have been frequently observed in multiple tumor tissues [14, 15], their functional impact remains controversial [16–18]. The most variable region in the T cell receptor and B cell receptor (TCR and BCR, respectively) is the complementarity-determining region 3 (CDR3), which plays a key role in antigen recognition [19, 20]. Therefore, characterizing tumor TCR and BCR repertoires, particularly the CDR3s, is critical to understanding antigen recognition and tumor–immune interactions. Efforts have been made to study the tumor-infiltrating TCR or BCR repertoires using either targeted deep sequencing (TCR-seq or BCR-seq) or unselected RNA-seq data in many solid tumors [21–24]. However, less is known about the immune repertoire changes in hematologic malignancies, and a systematic characterization of both TCR and BCR repertoires in the AML microenvironment is still lacking.

In this study, we characterized TCR and BCR repertoires in both pediatric and adult AML by detecting and analyzing the CDR3 sequences in TCR α, β, γ, and δ chains and B cell immunoglobulin (Ig) heavy (IgH) and light (IgL, IgK) chains from the RNA-seq data in AML patients and non-tumor donors. We investigated the clonal expansion patterns of T cells and B cells in the AML microenvironment and described the differences between AML and non-tumor samples. We also compared the differences between pediatric and adult AML samples and identified the association of tumor immune receptor repertoires with clinical outcome. These results provided insights into the immune receptor repertoires and T/B cell functions in AML.

## Methods

### In silico validation using single cell RNA-seq data

We previously developed a computational algorithm TRUST [22, 24–26] to extract TCR and BCR hypervariable CDR3 sequences from unselected bulk tumor RNA-seq data. In order to further validate the accuracy of our method for assembling TCR and BCR from RNA-seq data, we collected one SMART-seq dataset of CD45-positive white blood cells from 19 pre-treatment melanoma patients [27]. For each patient, we merged the single cell RNA-seq (scRNA-seq) data of the CD45-positive cells into one “bulk” sample and applied TRUST to extract the TCR/BCR reads as if it were regular RNA-seq data. In the single cell data, all the T/B cells have been identified based on known gene markers, providing the true fractions of T/B cells in each merged “bulk” sample. We then estimated the T/B cell fraction in each “bulk” sample using the number of reads mapped to TCR/BCR region from TRUST divided by the total number of sequencing reads. Moreover, we followed the instructions by Sade-Feldman et al. [27] to reconstruct T and B cell receptors from all the identified T and B cells. Only cells with unique sequence on both chains (e.g., it has been reported in [28] that some T cells have two different alpha chains) were counted in the downstream analysis of single cell data. In order to estimate the T/B cell clonotype diversity from single cell data, we calculated the Shannon entropy using the frequencies of TCR β chain and IgH CDR3 amino acid sequences. Samples with fewer than two single T/B cells were excluded in this analysis. In the simulated “bulk” data, we applied CPK (TCR/BCR CDR3s per kilo of TCR/BCR reads) [22] to estimate the clonotype diversity of T/B cells.

### Data collection and preprocessing

Our study investigated a total of 296 primary AML samples (Additional file 1: Table S1), including 145 pediatric samples from Therapeutically Applicable Research To Generate Effective Treatments (TARGET) [29] and 151 adult samples from The Cancer Genome Atlas (TCGA) [30]. The RNA-seq reads in BAM files, gene expression read counts, and clinical data of all the AML samples were downloaded from Genomic Data Commons (GDC, https://portal.gdc.cancer.gov/, Jun 2017). RNA-seq reads have been previously aligned to hg38 human reference genome using STAR2 [31] with the same parameters. As a control of the AML samples, RNA-seq data of 73 peripheral blood (PB) of non-tumor samples (Additional file 1: Table S2) were downloaded from Sequence Read Archive repository (SRA, https://www.ncbi.nlm.nih.gov/sra, PRJNA263846) and successfully processed using the GDC mRNA analysis pipeline (https://docs.gdc.cancer.gov/Data/Bioinformatics_Pipelines/Expression_mRNA_Pipeline). The limited available clinical annotation on these normal samples only allowed categorical information such as male/female and children/adults to be parsed out. Since the maturity of the adaptive immunity is dependent on age, especially in early age, the pediatric AML samples were further divided into infants (0–3 years old, *n* = 37) and children (3–20 years old, *n* = 108) group in the downstream analyses. Control samples were not divided due to the lack of age information.

### Detection and analysis of TCR and BCR CDR3 sequences from AML and non-tumor RNA-seq data

To characterize the immune receptor repertoires, we applied TRUST3.0.1 (https://bitbucket.org/liulab/trust) to all the AML and non-tumor RNA-seq samples. Formatted txt files with CDR3 calls were used in the downstream analyses, in which the est_lib_size column represents the number of reads mapped to TCR/BCR region. The number of total sequencing reads was obtained from each bam file using samtools [32], and those mapped to each variable (V), joining (J), or constant (C) genes were tallied in the “coverage.txt” file for each sample. The definition of the columns in these files was described in the TRUST documentation.

In order to compare the richness of TCR/BCR between AML and non-tumor samples, we normalized the number of CDR3s by the number of total sequencing reads and one minus blast percentage (pathologically estimated tumor purity) in each sample. The clonotype diversity of T/B cells was estimated by TCR/BCR CDR3s per kilo TCR/BCR reads (CPK) [22] in each sample. Complete CDR3 sequence was defined as CDR3 annotated with both V and J genes. γδ T cell fraction was estimated by the total number of γ or δ-CDR3s divided by the total number of TCR CDR3s in each sample.

To identify B cell lineage clusters in each sample, we extracted an octamer starting from the first position (not counting the starting "C") in each complete IgH CDR3 as motifs. All the IgH CDR3 sequences (either partial or complete) which contain amino acid matches to the motif with 0-1 mismatch (e.g., motifs RDMW**L**VGW and RDMW**I**VGW were considered matches) were collected. Each motif with 3 or more sequences was considered a B cell cluster. This approach provided flexibility in detecting amino acid changes from non-synonymous mutations, yet maintained low computational complexity.

Somatic hypermutation (SHM) [33] was defined as mismatches in B cell clusters. Mutations between two sequences with only one nucleotide mismatch were counted to avoid overestimation on SHM rate due the aggregated mutations during the B cell clonal expansion. SHM rate per sample was calculated as the SHM count divided by the total number of assembled CDR3 bases, which avoided the bias of unknown mutations outside partial CDR3 assembles. IgH CDR3 calls with unique isotype annotation were used in the isotype fraction and class switch recombination (CSR) analyses [34]. Cooccurrences of unambiguously assigned different Ig classes or subclasses in the same IgH CDR3 cluster were considered as CSR. The number of CSR events was normalized by the total number of IgH clusters in each group, and samples with less than 10 unique IgH CDR3s were excluded from downstream analyses.

### Statistical analysis

Wilcoxon rank-sum test was used to compare the differences between TCR/BCR CPK, γδ CDR3 fractions, and SHM rates among AML and non-tumor groups. Spearman’s rank correlation was used to check the association among αβ, γδ, or IgH and IgK/IgL CDR3 calls, and partial Spearman’s rank correlation was used to check the association between different Ig isotype fractions in the AML and non-tumor groups. Survival analyses were visualized using Kaplan–Meier curves, and the statistical significance was estimated using Log-rank test. Details for the other analyses were described in supplementary methods (Additional file 3).

## Results

### In silico validation on the accuracy of TRUST for assembling TCR and BCR CDR3s from RNA-seq data

The overall approach in our study has been repeatedly validated in our previous work [22, 24–26]. In this study, we applied the same approach to investigate the potential functional roles of T/B cells in AML using a large number of publicly available RNA-seq samples. Here, we also performed in silico validation on the accuracy of our method for assembling TCR and BCR from RNA-seq data by using publicly available scRNA-seq datasets on immune cells. We collected one SMART-seq dataset of CD45-positive white blood cells from pre-treatment melanoma patients [27]. Although these cells were derived from the infiltrating immune cells, they covered most of the cell types (macrophage, monocyte, dendritic cells, neutrophil, T/B lymphocytes, natural killer cells, etc.) composed of the AML immune microenvironment. We found that the fraction of both T and B cell estimated from single cell results and TRUST callings from “bulk” samples are significantly positively correlated (Additional file 2: Figure S1a). We then compared the associations of the number of TCR/BCR CDR3s between single cell data and TRUST callings from “bulk” samples. Again, they are also significantly positively correlated (Additional file 2: Figure S1b), indicating that the CDR3s detected by TRUST from bulk RNA-seq data provide a good approximation to the real T/B cell numbers in each sample. In order to estimate the T/B cell clonotype diversity from single cell data, we calculated the Shannon entropy using the frequencies of TCR β chain and BCR heavy chain CDR3 amino acid sequences. In the simulated “bulk” data, we applied CPK [22] to estimate the clonotype diversity of T/B cells. Consistently, we observed a significantly positive correlation between TCR/ BCR entropy and CPK (Additional file 2: Figure S1c). Based on these results and our previous work, we conclude that our approach has sufficient power to recover TCR and BCR CDR3s to evaluate the fraction and diversity of both T and B cells from bulk RNA-seq data, which allowed us to identify the changes of T and B cells between AML and non-tumor samples.

### Overview of TCR α, β, γ, and δ chain CDR3 sequences in AML and non-tumor samples

**Fig. 1:**
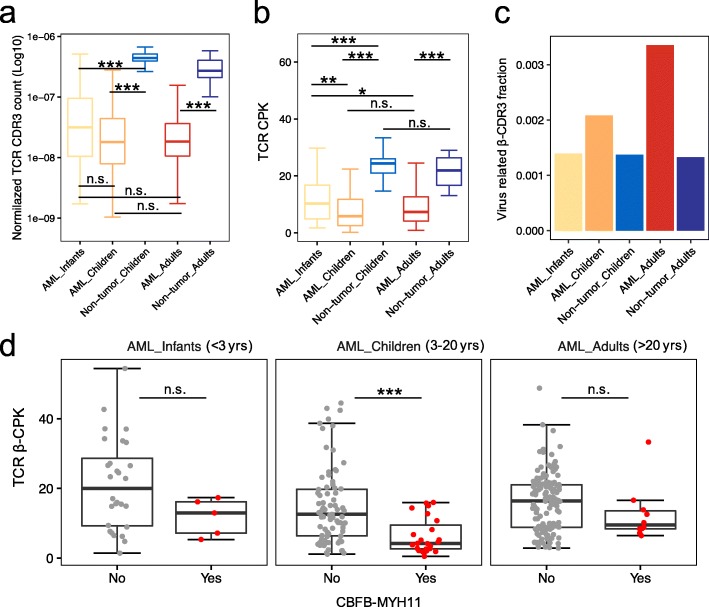
T cell diversity analysis in AML and non-tumor samples. **a** Normalized TCR CDR3 count in AML and non-tumor groups. The number of CDR3s was normalized by the number of total sequencing reads and one minus blast percentage (pathologically estimated tumor purity) in each sample. **b** TCR CDR3s per thousand (kilo) TCR reads (CPK, as a measure of clonotype diversity) in AML and non-tumor groups. **c** Barplot showing the ratio of virus-related β-CDR3 in AML and non-tumor groups. Virus-related β-CDR3 ratio was defined by the number of virus (cytomegalovirus, Epstein-Barr virus, and influenza)-related β-CDR3s divided by the total number of unique complete β-CDR3s in each group. **d** Comparison of β-CPK between samples with and without CBFB-MYH11 gene fusions. The *p* values in **a**, **b**, and **d** were calculated using the two-sided Wilcoxon rank-sum test. *p < 0.05, **p < 0.01, ***p < 0.001, n.s. indicates not significant

TRUST identified a total of 225,000 TCR CDR3 sequences from AML (55,000) and non-tumor samples (170,000). Despite deeper sequencing coverage of AML than non-tumor samples (Additional file 2: Figure S2a), we observed significantly fewer TCR CDR3 calls in AML (Additional file 2: Figure S2b), potentially due to the high malignant cell content in AML. In order to compare the richness of TCR between AML and non-tumor samples, we normalized the number of CDR3s by the sequencing depth and one minus blast percentage (pathologically estimated tumor purity) in each sample. As shown in Fig. 1a, the normalized TCR CDR3 counts are still significantly lower in AML samples. γδ chain CDR3s account for 5.8% of the total calls in AML and 6.6% in the non-tumor group (Additional file 2: Figure S2c), consistent with the previous estimation that γδ T cells constitute less than 10% of the total T cells in human PB [35]. In addition, we observed a positive correlation between α and β CDR3s and between γ and δ CDR3s from each sample in both AML and non-tumor groups (Additional file 2: Figure S2d, e), although we could not pair the αβ or γδ CDR3s with RNA-seq data. Overall, the length distribution of complete TCR α, β, γ, and δ chain CDR3s and their sequence conservation patterns are similar between the AML and non-tumor groups (Additional file 2: Figure S2f, g).

### The clonotype diversity of TCR repertoire in AML and non-tumor samples

T cell clonotype diversity is an important feature of the TCR repertoire which was previously reported to have potential clinical implications [36, 37]. We investigated the differences in T cell clonotype diversity between AML and non-tumor groups. Using CPK to approximate TCR clonal diversity [22], we observed significantly lower diversity in both pediatric and adult AML samples compared to non-tumor samples (Fig. 1b). This result suggests that T cells are more clonal in the AML microenvironment. No significant difference was observed in TCR diversity between PB and BM samples in the pediatric AML (Additional file 2: Figure S3a) or between pediatric and adult non-tumor samples (Fig. 1b). Interestingly, we found that infant AML samples have significantly higher TCR CPK than children or adult AML (Fig. 1b). This result suggests that T cells are less expanded in infant AML, which might be due to limited bacterial and viral antigen exposure during infancy. Consistently, we also observed lower fraction of β-CDR3s specific to common viral epitopes from cytomegalovirus, Epstein–Barr virus, or influenza [38], in infant AML than in children or adult AML (Fig. 1c).

Neo-antigens arising from somatic mutations can induce T cell-mediated elimination of cancer cells [39]. A direct consequence of antigen-specific T cell activation is clonal expansion, which can be approximated by the inverse of CPK. We therefore sought to investigate whether specific missense mutation or gene fusion, which has been linked to patient survival, was associated with αβ T cell activation in AML samples. Due to the lack of detailed mutation information from pediatric AML samples, we could only check the mutation status available on five genes with high clinical relevance (FLT3, NPM1, KIT, CEBPA, and WT1) and on three oncogenic gene fusions (RUNX1-RUNX1T1, CBFB-MYH11, and PML-RARA). We found that pediatric AML samples with CBFB-MYH11 fusions have significantly lower TCRβ CPK value (Fig. 1d), suggesting this fusion as potentially immunogenic. The same trend was also observed in infant and adult AML, although the difference is not as significant due to the limited sample size.

### γδ T cell analysis in AML and non-tumor samples

**Fig. 2:**
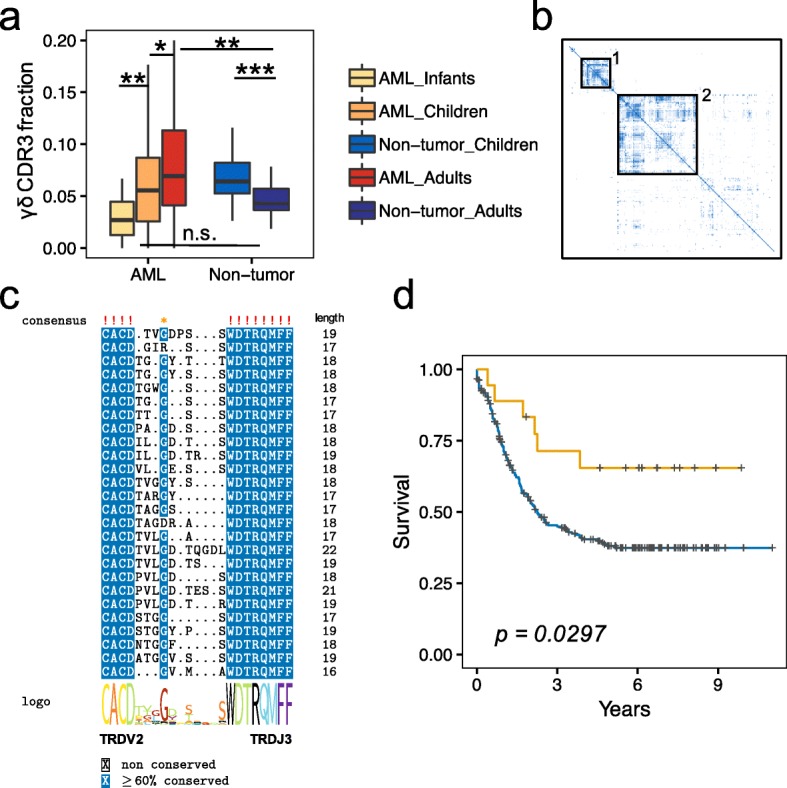
γδ T cell analysis in AML and non-tumor samples. **a** γδ T cell fraction in AML and non-tumor groups. γδ T cell fraction was estimated by the summed number of γ or δ-CDR3s divided by the number of total TCR CDR3s in each sample. The *p* values were calculated using the two-sided Wilcoxon rank-sum test. *p < 0.05, **p < 0.01, ***p < 0.001, n.s. indicates not significant. **b** Heatmap of δ-CDR3 amino acid sequences similarity matrix. Local alignment and BLOSUM62 were used to calculate the similarity between each pair of the complete δ-CDR3 amino acid sequences. Blue color indicates high similarity. **c** Sequence motif analysis of δ-CDR3s in Cluster1. **d** Kaplan–Meier curves showing AML samples with δ-CDR3 belonging to the Cluster1 have better overall survival (*n* = 19, yellow line, *p* value was evaluated using Log-rank test)

γδ T cells constitute a small percentage of total T cells in human PB, and their roles in antitumor immune responses have not been well characterized. Although the fraction of γδ CDR3s are similar between AML and non-tumor samples (Fig. 2a) and between PB and BM samples in pediatric AML (Additional file 2: Figure S3b), there are intriguing age-related differences. In the non-tumor group, the fraction of γδ CDR3s is higher in children compared to adults (Fig. 2a), which is consistent with the previous report that γδ T cell frequency and diversity decrease with age [40]. In contrast, the opposite was observed in AML where the fraction of γδ CDR3s increases with age (Fig. 2a). A recent study reported that Vγ9Vδ2 T cells are able to recognize and kill AML blasts through a TCR-dependent manner [41]. Together with our observations, this suggests that since γδ T cells could interact with and eradicate AML blasts, leukemic cells might alter γδ T cell development or distribution in AML.

To further investigate the potential impact of γδ T cells in AML, we clustered all the complete δ-CDR3s based on their pairwise sequence similarity. This revealed two major clusters of the δ-CDR3 sequences (Fig. 2b), with Cluster1 containing 26 sequences from 19 patients. All the δ-CDR3s in Cluster1 were annotated to be associated with TRDV2 and TRDJ3. Sequence motif analysis of Cluster1 δ-CDR3s revealed the first 4 and last 8 amino acids to be conserved (Fig. 2c), as well as a glycine (G) in the middle. Intriguingly, these individuals have significantly better overall survival (Fig. 2d) compared to the other patients. These results suggest that the δ-CDR3s containing the specific pattern in Cluster1 might serve as a potential prognosis marker or potential therapeutic target for AML patients.

### Overview of BCR IgL, IgK, and IgH CDR3 sequences in AML and non-tumor samples

**Fig. 3:**
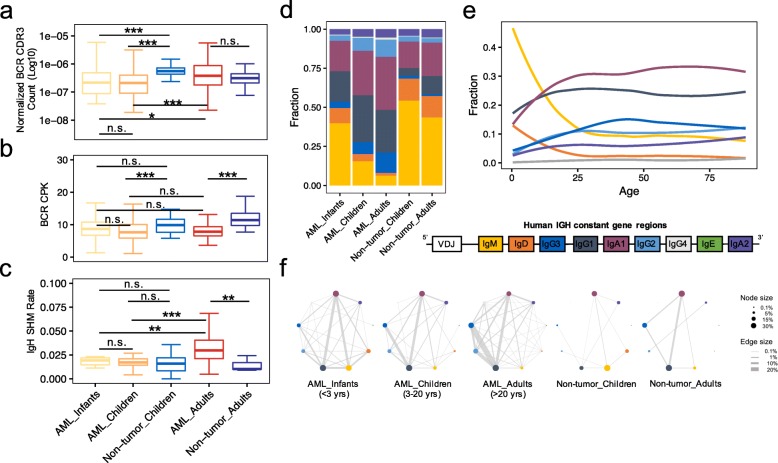
The abnormal activation of B cells in AML samples. **a** Normalized BCR CDR3 count in AML and non-tumor groups. The number of CDR3s was normalized by the number of total sequencing reads and one minus blast percentage (pathologically estimated tumor purity) in each sample. **b** BCR CPK in AML and non-tumor groups. **c** IgH SHM rate in AML and non-tumor groups. The *p* values in **a**, **b**, and **c** were calculated using the two-sided Wilcoxon rank-sum test. *p < 0.05, **p < 0.01, ***p < 0.001, n.s. indicates not significant. **d** Distribution of 9 Ig isotypes across AML and non-tumor groups. **e** The regression curves of 8 Ig isotype fractions against age in AML samples. IgE was excluded due to the extremely low fraction in most samples. **f** Visualization of Ig isotype class switching in AML and non-tumor groups. Circle size represents the fraction of Ig isotypes, which is the number of IgH clusters carrying a given Ig isotype divided by the total number of IgH clusters in each group. Lines connecting two circles indicate co-existence of two isotypes in one cluster, with line width proportional to the number of such cluster divided by the total number of IgH clusters in each group

We next investigated the changes of BCR repertoires in the AML microenvironment. TRUST derived a total of 1,210,000 BCR (IgL, IgK, and IgH) CDR3s from the AML (974,000) and non-tumor (236,000) samples (Additional file 2: Figure S4a). Similar to the lower number of TCR CDR3 calls in AML, the number of BCR CDR3 calls is also significantly lower in the AML samples compared to non-tumor samples (Fig. 3a, Additional file 2: Figure S4b). In addition, the number of Ig light chain (IgL and IgK) and Ig heavy chain (IgH) CDR3s from each sample, despite not paired, is significantly positively correlated in both AML and non-tumor groups (Additional file 2: Figure S4c). There is no significant difference in IgL to IgK CDR3 ratio between AML and non-tumor samples (Additional file 2: Figure S4d) or between PB and BM samples in pediatric AML (Additional file 2: Figure S5a). However, IgL to IgK ratio is significantly lower in adult than in pediatric samples in both AML and non-tumor groups (Additional file 2: Figure S4d), indicating the age-related difference in IgL vs IgK usage. The length distribution of complete IgL and IgK CDR3s and their sequence conservation patterns are similar between the AML and non-tumor groups (Additional file 2: Figure S4e, f). In contrast, complete IgH CDR3s are significantly longer in AML than in non-tumor samples (Additional file 2: Figure S4e, IgH), as well as in PB than in BM samples in pediatric AML (Additional file 2: Figure S5b). We previously reported IgH CDR3 sequences from expanded tumor-infiltrating B cell clones to be significantly longer than the non-expanded clones in solid tumors [24]. Thus, the longer IgH CDR3s we observed in AML might be a consequence of the higher level of B cell clonal expansion in the AML microenvironment.

### B cell activation and clonal expansion patterns in the AML samples

We further investigated the potential impact of B cells in AML. Similar to the lower TCR diversity, BCR CDR3 diversity in terms of CPK is also lower in AML samples than in non-tumor samples (Fig. 3b). Unlike T cells, B cells, upon binding to a foreign antigen, undergo SHM and CSR to produce high affinity antibodies against the antigen. Therefore, SHM and CSR are important signatures of B cell activation and clonal expansion. To investigate SHM rate, we counted the cases where two IgH CDR3 sequences differ by only one nucleotide, and divided the count by the total assembled CDR3 bases in each sample. Using this measure, we observed significantly higher SHM rate in adult AML samples compared to pediatric AML samples or non-tumor samples (Fig. 3c). Consistent with this result, AICDA [42], the gene responsible for SHM, also has significantly higher expression in the adult AML samples compared to pediatric AML samples (Additional file 2: Figure S6). To investigate CSR, we examined the approximately 346,000 IgH sequences that were successfully aligned to specific Ig isotypes. We observed significant differences in the isotype distributions among AML and non-tumor groups (Fig. 3d). Specifically, in the non-tumor samples, IgM and IgD, which are the first two heavy chain constant segments in the immunoglobulin locus and usually expressed on naïve mature B cells [43], account for the majority of the total IgH sequences (Fig. 3d). Infant AML samples also have higher IgM and IgD B cells, but as AML patients age, the fraction of IgG and IgA increase (Fig. 3e). IgG1 and IgA1 become the dominant Ig isotypes in children and adult AML samples (Fig. 3d, e). When normalizing against the expression of housekeeping genes, we found that the level of IgM and IgD only decreased slightly, suggesting that the increase of IgG and IgA fraction is mostly due to the expansion of B cells with IgA and IgG isotypes (Additional file 2: Figure S7). In addition, AML samples show more CSR events than non-tumor samples (Fig. 3f). Taken together, the increased IgH CDR3 length, decreased IgH CDR3 diversity, increased SHM, and increased CSR in AML, especially with IgG and IgA isotypes in adult AML, all indicate higher levels of B cell activation and clonal expansion in the AML microenvironment.

### Association between high IgA fraction and worse clinical survival in AML patients

**Fig. 4:**
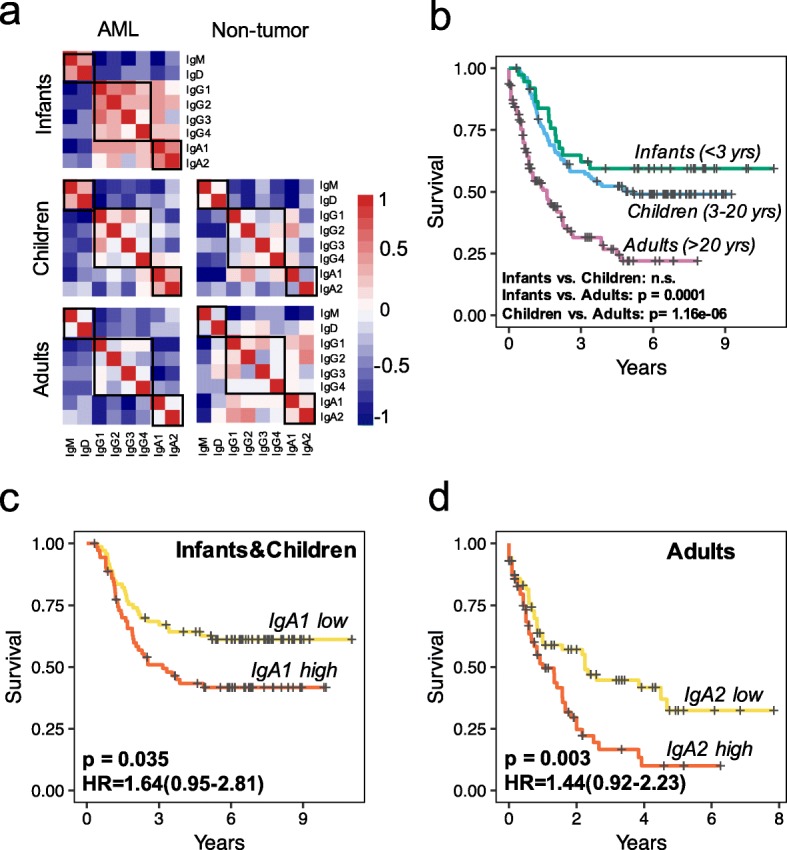
Worse clinical outcome for AML samples with high fraction of IgA1 or IgA2. **a** Heatmaps showing the correlations of different Ig isotype fractions in AML and non-tumor groups. Partial Spearman’s rank correlation was used to check the association between different Ig isotypes. Correlation coefficient, controlling for age, was shown in heatmaps for AML and non-tumor groups. **b** Kaplan–Meier curves showing the survival difference among infant, children, and adult AMLs. Infants and children showed better overall survival compared to adults, without significant difference between the two groups. Statistical significance comparing different groups was evaluated using Log-rank test. **c**, **d** Kaplan–Meier curves showing the pediatric AML samples with high IgA1 fraction (**c**) and the adult AML samples with high IgA2 fraction (**d**) have worse overall survival. Samples were divided into IgA1 (or IgA2) ratio high and IgA1 (or IgA2) ratio low group by the median fraction of this ratio in pediatric/adult AMLs. The IgA1 (or IgA2) ratios were calculated using the number of IgA1 (or IgA2) CDR3s divided by the total number of IgH CDR3s with unique Ig class annotation in each sample. Statistical significance comparing different groups was evaluated using multivariate Cox regression corrected for patient gender and age at diagnosis

The abnormal activation of IgA and IgG B cells in the AML microenvironment prompted us to examine their association with clinical features. IgA can be divided into IgA1 and IgA2 subclasses, while IgG isotype can be further divided into IgG1, IgG2, IgG3, and IgG4 subclasses. Although different subclasses share high sequence similarity, they still have different heavy chain structures and distinct effector functions [44]. Although different IgA subclasses or IgG subclasses are highly correlated in infant AML, subclass correlation is lower in children AML and even lower in adult AML (Fig. 4a). In addition, significant differences in patients’ overall survival exist between pediatric and adult AML (Fig. 4b). We thus examined the impact of different IgA and IgG subclasses on pediatric and adult patients’ survival separately. No significant association was observed between IgG isotype or subclasses and patients’ overall survival. However, we found that pediatric AML patients with higher fraction of IgA1 (Fig. 4c) and adult AML patients with higher fraction of IgA2 (Fig. 4d) have significantly worse overall survival (Additional file 2: Figure S8). Higher IgA ratio has been reported to be associated with worse clinical outcome in melanoma [45]. Therefore, our observation of IgA association with worse clinical outcome suggests that IgA B cells might be associated with a suppressive immune microenvironment in AML.

### IgA2 fraction and immunosuppressive microenvironment in adult AML

**Fig. 5:**
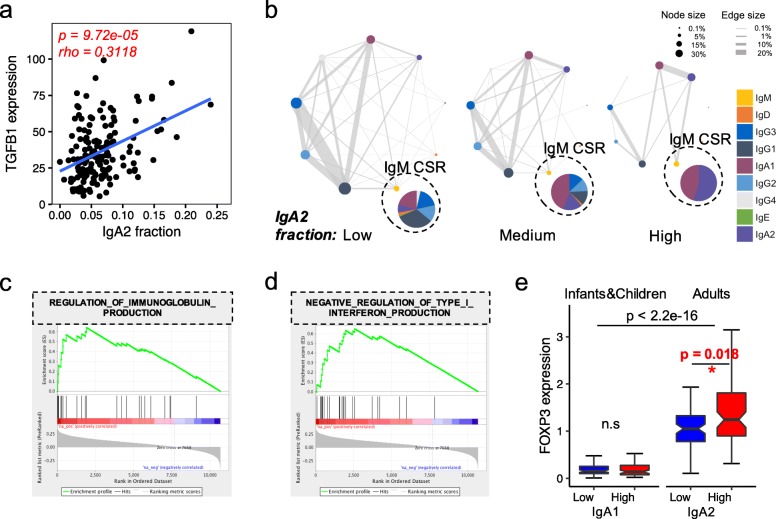
High fraction of IgA2 associated with immunosuppressive microenvironment in adult AML. **a** Scatter plot showing the positive correlation between IgA2 fraction and TGFB1 expression in adult AML. Statistical significance was evaluated using Spearman’s correlation test. **b** Visualization of Ig isotype class switching in adult AML groups. Adult AML samples were divided into IgA2 low (0–5%, *n* = 64), medium (5%–10%, *n* = 64), and high (> 10%, *n* = 23) groups. Circle size represents the fraction of Ig isotypes, which was calculated by the number of IgH clusters carrying a given Ig isotype divided by the total number of IgH clusters in each group. Lines connecting two circles indicate co-existence of two isotypes in one cluster, with line width proportional to the number of such cluster divided by the total number of IgH clusters in each group. Network size represents the overall B cell activation, which is defined by the number of IgH CDR3 clusters divided by the number of IgH CDR3s in each group. The pie charts in black dot circles show the fraction of IgM class switching across different groups. **c**, **d** The enriched GO terms with IgA2 fraction in adult AML. **e** Boxplot showing FOXP3 expression level across pediatric AML IgA1 low, high, and adult AML IgA2 low, high groups. The *p* values were calculated using the two-sided Wilcoxon rank-sum test

Recent mouse studies reported that TGFβ-induced IgA-producing plasma cells can function as potent immunosuppressors through the secretion of PD-L1 [46, 47]. Consistent with these reports, in adult AML samples, we observed a significantly positive correlation between TGFB1 expression and IgA2 fraction (Fig. 5a). In AML samples with higher IgA2, besides having a lower level of IgG (Additional file 2: Figure S9) which is known to promote T cell-mediated antitumor immunity [48], the CSR events of IgM B cells are almost restricted to IgA1 and IgA2 (Fig. 5b). Moreover, GSEA [49] analysis revealed that genes positively correlated with IgA2 in adult AML are significantly enriched in the negative regulation of type I interferon production (Fig. 5c, d, Additional file 2: Figure S10) which is an important regulator of innate and adaptive immune responses [50]. To evaluate whether PD-L1 is the downstream effector of TGFβ and IgA production, we further examined whether IgA2 high AML tumors also have higher PD-L1 expression, but found no significant difference (Additional file 2: Figure S11). Instead, in the IgA2 high AML tumors, the expression of the regulatory T cell (Treg) marker FOXP3 is significantly higher (Fig. 5e). This suggests that Treg recruitment might be an alternative mechanism of TGFβ/IgA-induced immunosuppression which contributes to the worse overall survival in adult AML.

## Discussion

AML is a common hematologic malignancy, although the interactions between malignant myeloid cells and the immune microenvironment, especially T cells and B cells, remain poorly characterized. In this study, we conducted the first comprehensive characterization of TCR (α, β, γ, and δ chains) and BCR (IgL, IgK, and IgH) CDR3 from the bulk RNA-seq data from both pediatric and adult AML samples as well as non-tumor controls. The human immune system evolves with age, as exposures to multiple self and foreign antigen challenges promote the maturation of immune-related cells and organs [40]. We found higher clonal expansion of both T cells and B cells in the AML microenvironment, but observed wide differences between pediatric and adult AML. In particular, we found that adult AML samples have higher fraction of γδ T cells (Fig. 2a) and higher level of IgH SHM rate and CSR events compared to pediatric AML (Fig. 3). One limitation of our study is that we do not have age information for the non-tumor samples, so we could not analyze the age effect in normal donors, although this does not bias any of our findings. Another limitation of this work is that due to the use of bulk RNA-seq data, it is not possible to match the full clonal type (TCR αβ, γδ chain, and BCR heavy light chain) or distinguish subtypes of T and B cells in our analysis. Despite these limitations, our findings help improve our understanding of T and B cell immunity in AML as well as the distinct immune responses of T cells and B cells to AML between children and adults. Our results might provide insights into immunotherapy development in hematological malignancies.

Notably, we found that pediatric AML with highly expanded IgA1 B cells and adult AML with highly expanded IgA2 B cells, which might represent an immunosuppressive microenvironment, are associated with worse overall survival. Recent studies reported that IgA-producing plasma cells can function as potent immunosuppressors through the secretion of PD-L1 in prostate [46] and liver cancer mouse models [47]. Unlike mouse IgA which has only one subclass, human IgA comprises two subclasses (IgA1 and IgA2) encoded by two distinct genes. The lack of elongated hinge regions in IgA2 Fc ligand forms the major structure difference between the two subclasses [51]. We found the survival-related B cells are restricted to IgA1 in pediatric but to IgA2 in adult AML samples (Fig. 4c, d). Together with many differences observed between pediatric and adult AML, we interpret this as potentially related to the different immune response patterns between children and adults. The IgA CSR is known to be related to the secreted cytokine TGFβ1 [52], and we observed a significant positive correlation between TGFB1 gene expression and IgA2 fraction in adult AML (Fig. 5a). In addition, in a single cell expression data from one M6 AML patient [53], we found TGFB1 to be highly expressed in three major cell clusters, including CD4+CD14+ monocytes, PRSS57+MYC+ neutrophils, and CD3+CD7+ T cells (Additional file 2: Figure S12), suggesting a complex regulation of IgA2 B cell proliferation in AML. Our findings may shed light on the unique immune regulation in hematological malignancies.

## Conclusions

In summary, our comprehensive analyses of TCR and BCR CDR3 sequences from AML RNA-seq samples provided the first overview of the immune receptor repertoires in both pediatric and adult AML microenvironments. We found a higher clonal expansion of both T cells and B cells in the AML microenvironment. In addition, adult AML samples have a significantly higher level of B cell activation and more secondary Ig class switch events than pediatric AML or non-tumor samples. Furthermore, we found that pediatric AML with highly expanded IgA1 B cells and adult AML with highly expanded IgA2 B cells are associated with worse overall survival. The identified TCR/BCR repertoires and the observed associations from this work provide useful resources and insights into the future development of novel immunotherapies for hematological malignancies.

## Supplementary information

**Additional file 1: Table S1.** Clinical characteristics of AML samples. **Table S2.** Clinical characteristics of non-tumor samples.

**Additional file 2: Figure S1.** In silico validation on the accuracy of TRUST for assembling TCR and BCR from RNA-seq data by using single cell RNA-seq data of CD45 positive white blood cells from pre-treatment melanoma patients. **Figure S2.** Overview of TCR CDR3s in AML and non-tumor samples. **Figure S3.** Comparison of TCR CPK and γδ T cell fraction between PB and BM samples in pediatric AML. **Figure S4.** Overview of BCR CDR3s in AML and non-tumor samples. **Figure S5.** Comparison of BCR CDR3s between PB and BM samples in pediatric AML. **Figure S6.** Violin plot showing the expression level of AICDA is significantly higher in adult AML than pediatric AML. **Figure S7.** Boxplots showing the normalized number of IgA/IgG/IgM + IgD CDR3s in AML and non-tumor groups. **Figure S8.** Kaplan-Meier curves showing the survival difference relative to IgA fraction in pediatric and adult AML. **Figure S9.** Distribution of 9 Ig isotypes across adult AML IgA2 low (0–5%, *n* = 64), medium (5%–10%, n = 64) and high (> 10%, *n* = 23) group. **Figure S10.** The enriched GO terms with IgA2 fraction in adult AML. **Figure S11.** Boxplot showing the expression level of PD-L1 in pediatric AML IgA1 high/low group and adult AML IgA2 high/low group. **Figure S12.** t-SNE plots showing the expression level of different gene makers in normal and AML (M6, one patient) peripheral blood single cell RNA-seq data.

**Additional file 3.** Supplementary methods.

**Additional file 4.** Processed data for both AML and non-tumor samples.

## Data Availability

The results published here are in whole or part based upon the data generated by the TARGET (https://ocg.cancer.gov/programs/target) initiative, phs000465. The TARGET and TCGA AML datasets [29, 30] analyzed during the current study are available in the Genomic Data Commons (GDC, https://portal.gdc.cancer.gov/). The RNA-seq FASTQ files of non-tumor blood samples are available in Sequence Read Archive repository (SRA, https://www.ncbi.nlm.nih.gov/sra), under BioProject accession code PRJNA263846. The derived TCR and BCR CDR3 sequences and the HLA information for each sample are available from FireCloud (https://portal.firecloud.org) with the corresponding dbGap access right. The processed data for both AML and non-tumor samples are available in Additional file 4.

## Abbreviations

AML: Acute myeloid leukemia
BCR: B cell receptor
BM: Bone marrow
CAR: Chimeric antigen receptor
CDR3: Complementarity-determining region 3
CPK: Clonotypes per kilo reads
CSR: Class switch recombination
GDC: Genomic Data Commons
ICB: Immune checkpoint blockade
Ig: Immunoglobulin
IgH: Immunoglobulin heavy chain
IgK: Immunoglobulin kappa light chain
IgL: Immunoglobulin lambda light chain
PB: Peripheral blood
SHM: Somatic hypermutations
TARGET: Therapeutically Applicable Research To Generate Effective Treatments
TCGA: The Cancer Genome Atlas
TCR: T cell receptor
Treg: Regulatory T cells
