# Supplementary material for: Immune receptor repertoires in pediatric and adult acute myeloid leukemia
Source: Genome Med. 2019 Nov 26;11:73. doi: 10.1186/s13073-019-0681-3 (PMC6880565; doi:10.1186/s13073-019-0681-3)
Supplement: Supplementary file 1 — Additional file 1: Table S1. Clinical characteristics of AML samples. Table S2. Clinical characteristics of non-tumor samples. [file 13073_2019_681_MOESM1_ESM.pdf]

Table S1. Clinical characteristics of AML samples

Characteristic	AML dataset	
	TARGET	TCGA
Age	9.2±6.1	55.0±16.1
Gender(male)	74(51.7%)	83(54.6%)
Race or ethnic group		
White	112(76.2%)	135(89.4%)
Black or African American	15(10.3%)	13(8.6%)
Other	12(8.3%)	1(0.7%)
Unknown	6(4.1%)	2(1.3%)
Leukemic blast percent	71.2±21.4	69.3±19.1
Cytogenetic risk group		
Favorable	60(41.4%)	31(20.5%)
Intermediate	69(47.6%)	82(54.3%)
Unfavorable	8(5.5%)	36(23.8%)
Missing data	8(5.5%)	2(1.3%)
AML FAB Category		
AML with minimal maturation: M0	3(3.1%)	15(9.9%)
AML without maturation: M1	17(11.7%)	35(23.2%)
AML with maturation: M2	35(24.1%)	38(25.2%)
Acute promyelocytic leukemia: M3	0	15(9.9%)
Acute myelomonocytic leukemia: M4	36(24.8%)	29(19.2%)
Acute monoblastic or monocytic leukemia: M5	30(20.7%)	15(9.9%)
Acute erythroid leukemia: M6	2(1.4%)	2(1.3%)
Acute megakaryoblastic leukemia: M7	7(4.8%)	1(0.7%)
Not classified	15(10.3%)	1(0.7%)
Sample type		
Peripheral blood (PB)	26(17.9%)	151(100%)
Bone marrow (BM)	119(82.1%)	
Total	145	151

Table S2. Clinical characteristics of non-tumor samples

**Characteristic**	**Non-tumor dataset**	
	Children	Adults
Age	NA	NA
Gender(male)	22(39.3%)	8(47.1%)
Race or ethnic group	NA	NA
Sample type	PB	PB
Total	56	17
