# Supplementary material for: Immune receptor repertoires in pediatric and adult acute myeloid leukemia
Source: Genome Med. 2019 Nov 26;11:73. doi: 10.1186/s13073-019-0681-3 (PMC6880565; doi:10.1186/s13073-019-0681-3)
Supplement: Supplementary file 2 — Additional file 2: Figure S1. In silico validation on the accuracy of TRUST for assembling TCR and BCR from RNA-seq data by using single cell RNA-seq data of CD45 positive white blood cells from pre-treatment melanoma patients. Figure S2. Overview of TCR CDR3s in AML and non-tumor samples. Figure S3. Comparison of TCR CPK and γδ T cell fraction between PB and BM samples in pediatric AML. Figure S4. Overview of BCR CDR3s in AML and non-tumor samples. Figure S5. Comparison of BCR CDR3s between PB and BM samples in pediatric AML. Figure S6. Violin plot showing the expression level of AICDA is significantly higher in adult AML than pediatric AML. Figure S7. Boxplots showing the normalized number of IgA/IgG/IgM + IgD CDR3s in AML and non-tumor groups. Figure S8. Kaplan-Meier curves showing the survival difference relative to IgA fraction in pediatric and adult AML. Figure S9. Distribution of 9 Ig isotypes across adult AML IgA2 low (0–5%, n = 64), medium (5%–10%, n = 64) and high (> 10%, n = 23) group. Figure S10. The enriched GO terms with IgA2 fraction in adult AML. Figure S11. Boxplot showing the expression level of PD-L1 in pediatric AML IgA1 high/low group and adult AML IgA2 high/low group. Figure S12. t-SNE plots showing the expression level of different gene makers in normal and AML (M6, one patient) peripheral blood single cell RNA-seq data. [file 13073_2019_681_MOESM2_ESM.pdf]

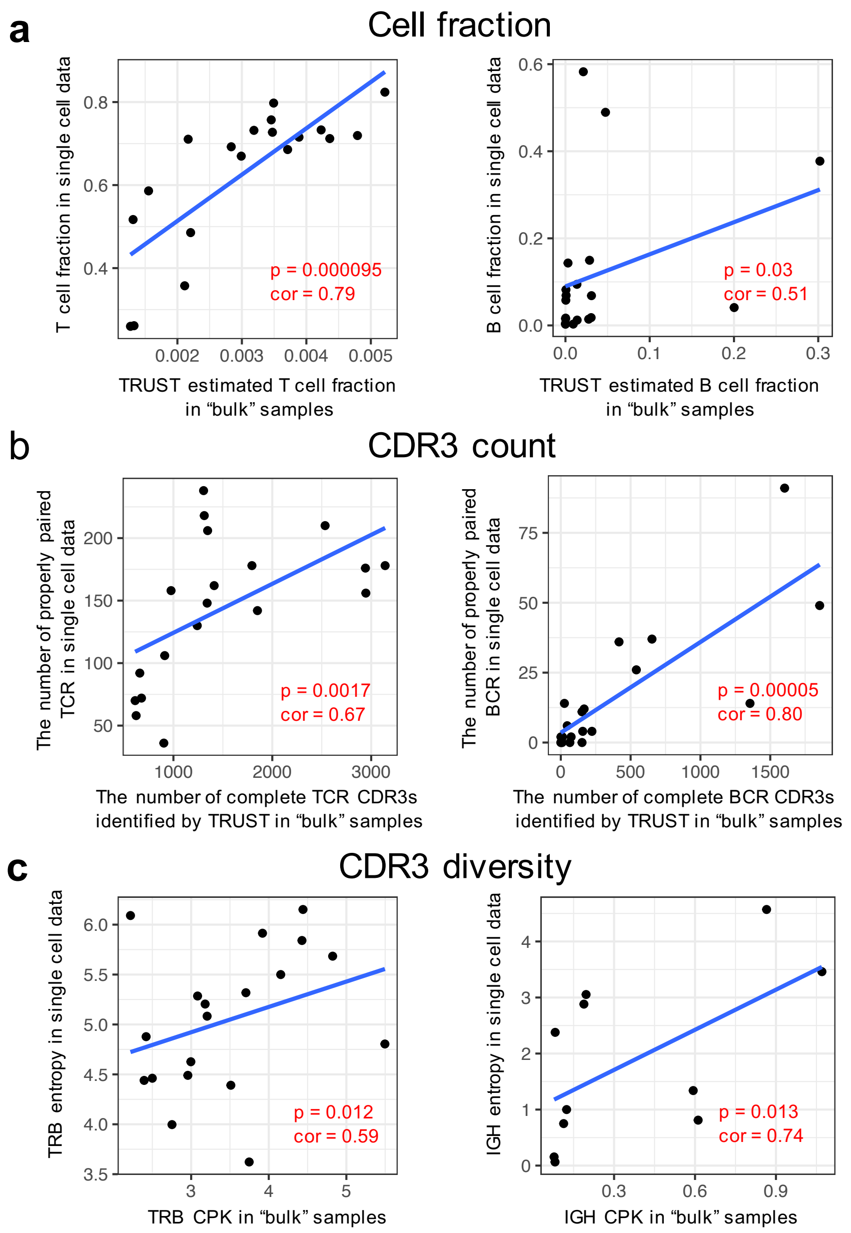

Fig. S1. *In silico* validation on the accuracy of TRUST for assembling TCR and BCR from RNA-seq data by using single cell RNA-seq data of CD45 positive white blood cells from pre-treatment melanoma patients. **a)** Scatter plot showing the positive correlation of T/B cell fraction between single cell results and TRUST callings from "bulk" samples. **b)** Scatter plot showing the positive correlation of the number of TCR/BCR CDR3s between single cell results and TRUST callings from "bulk" samples. **c)** Scatter plot showing the positive correlation between TCR/ BCR entropy and CPK (TCR/BCR CDR3s per kilo of TCR/BCR reads). Eight samples with fewer than two single T/B cell were excluded in this analysis.

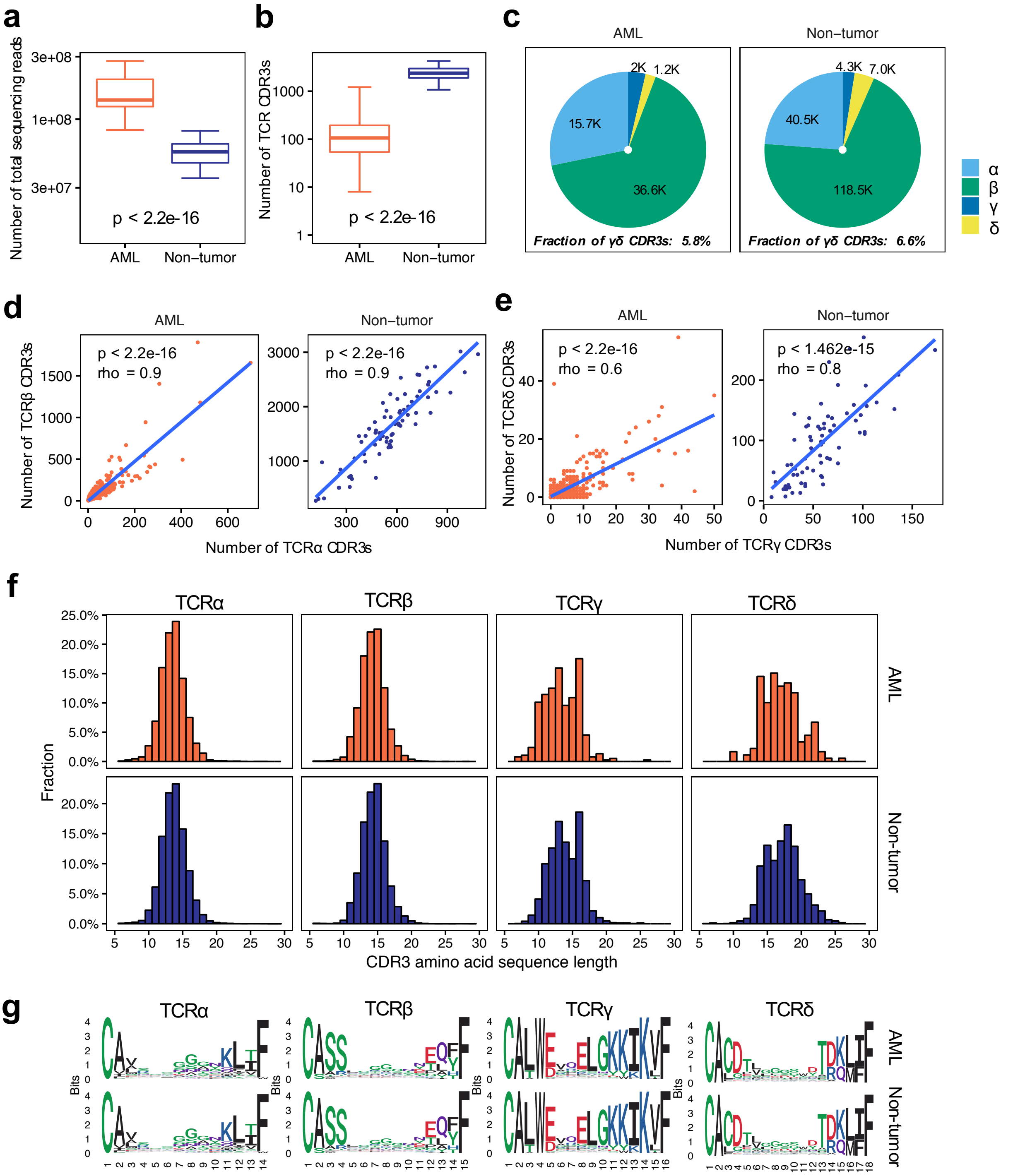

Fig. S2. Overview of TCR CDR3s in AML and non-tumor samples. **a)** Comparison of the number of total sequencing reads between AML and non-tumor group. **b)** Comparison of the number of TCR CDR3 calls between AML and non-tumor group. The p values in **(a, b)** were calculated using the two-sided Wilcoxon rank-sum test. **c)** Pie charts showing the fraction of TCR α, β, γ, δ CDR3s in AML and non-tumor group. **d, e)** Scatter plot showing the positive correlation between α and β CDR3s **(d)** and between γ and δ CDR3s **(e)** in AML and non-tumor group. Statistical significance in **(d, e)** were evaluated using Spearman's correlation test. **f)** Histograms showing the length distributions of complete TCR CDR3 amino acid (AA) sequences. **g)** The sequence logos are for all the CDR3s with the highest frequency in length (TCRα 14AA, β 15AA, γ 16AA and δ 18AA) in each group.

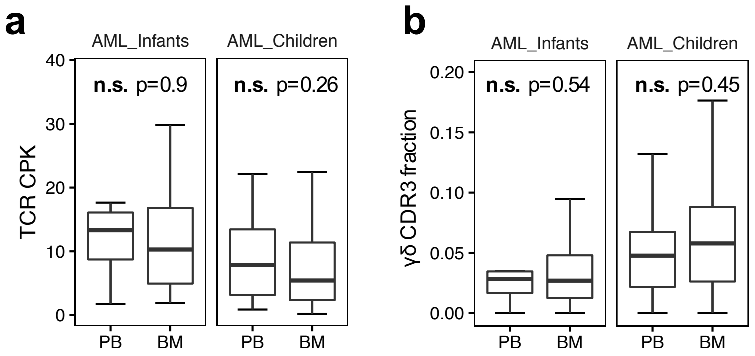

c Pediatric AML

	**PB**	**BM**
Infants	4	33
Children	22	86

Fig. S3. Comparison of TCR CPK and γδ T cell fraction between PB and BM samples in pediatric AML. **a)** Boxplot showing there are no significant difference of TCR CPK between PB and BM samples. **b)** Boxplot showing there are no significant difference of γδ T cell fraction between PB and BM samples. γδ T cell fraction was estimated by the summed number of γ or δ-CDR3s divided by the number of total TCR CDR3s in each sample. The p values were calculated using two-sided Wilcoxon rank-sum test. **c)** The number of PB and BM samples in each group.

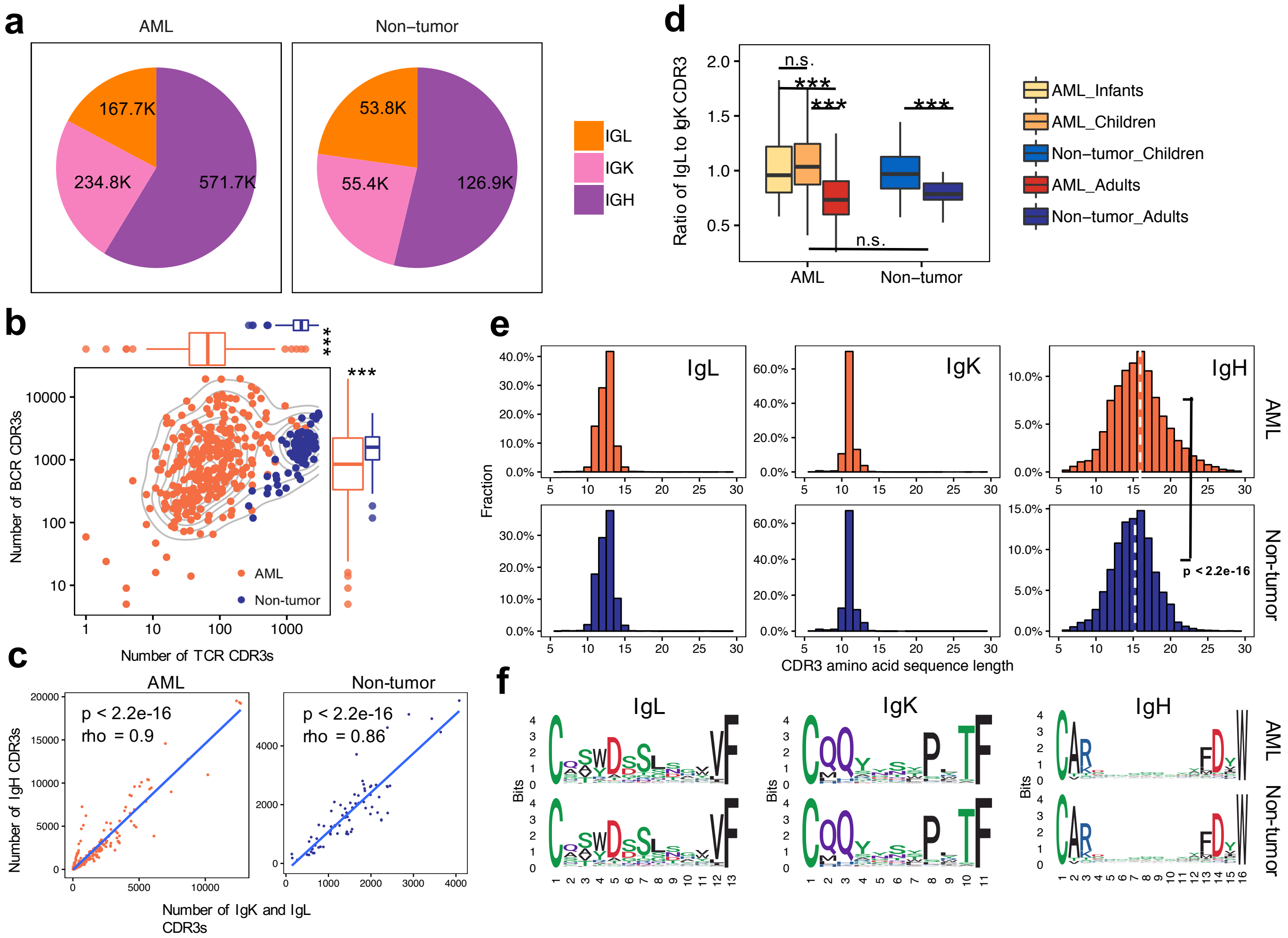

Fig. S4. Overview of BCR CDR3s in AML and non-tumor samples. **a)** Pie charts showing the fraction of IgL, IgK, and IgH CDR3 calls in AML and non-tumor group. **b)** Density plot showing the number of TCR CDR3s and the number of BCR CDR3s detected from AML and non-tumor samples. The boxplot plots on the top/right showing the difference of TCR/BCR CDR3 count between AML and non-tumor group. **c)** Scatter plot showing the positive correlation between IgL/IgK and IgH CDR3 calls in AML and non-tumor group. Statistical significance were evaluated using Spearman's correlation test. **d)** The ratio of IgL to IgK CDR3s in AML and non-tumor groups. The p values in **(b, d)** were calculated using the two-sided Wilcoxon rank-sum test. * p<0.05, **p<0.01, ***p<0.001, n.s. indicates not significant. **e)** Histograms showing the length distributions of complete BCR CDR3 amino acid (AA) sequences. White dashed lines indicate the median length of complete IgH CDR3s in AML and non-tumor group. The p value was calculated using Student's *t*-test. **f)** The sequence logos are for all the CDR3s with the highest frequency in length (IgL 12AA, IgK 11AA, and IgH 16AA) in each group.

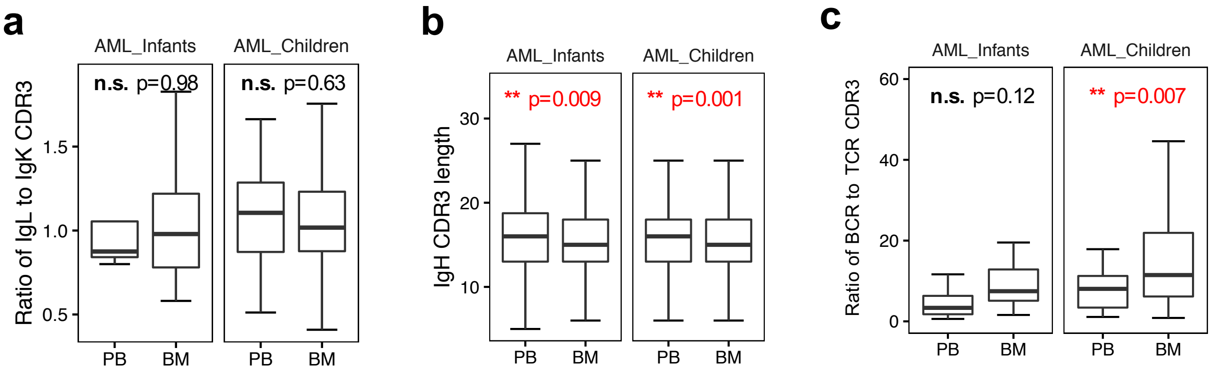

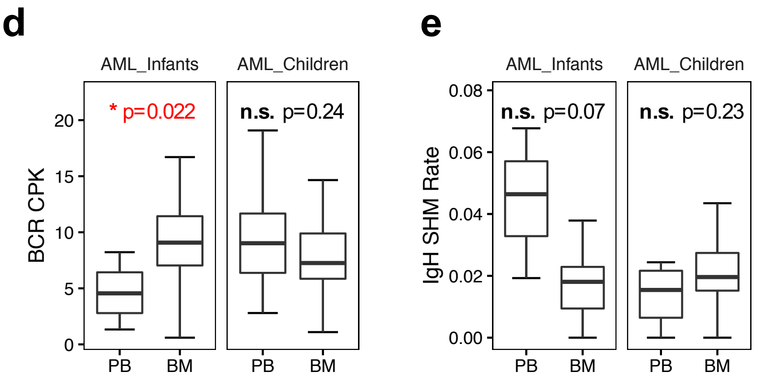

f

Pediatric AML

	PB	BM
Infants	4	33
Children	22	86

Fig. S5. Comparison of BCR CDR3s between PB and BM samples in pediatric AML. Boxplot showing the ratio of IgL to IgK CDR3s **(a)**, complete IgH CDR3 length **(b)**, the ratio of BCR to TCR CDR3s **(c)**, BCR CDR3 diversity **(d)**, and IgH SHM rate **(e)** between PB and BM samples. The p values in **(a-e)** were calculated using the two-sided Wilcoxon rank-sum test. * $p<0.05$, ** $p<0.01$, *** $p<0.001$, n.s. indicates not significant.

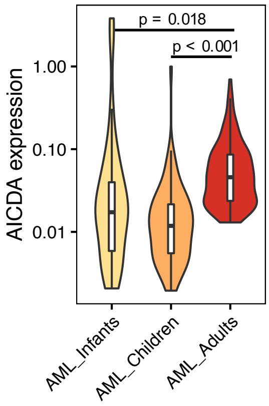

Fig. S6. Violin plot showing the expression level of AICDA is significantly higher in adult AML than pediatric AML. The p values were calculated using the two-sided Wilcoxon rank-sum test.

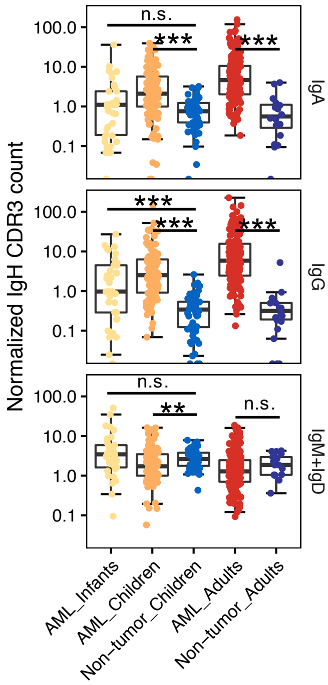

Fig. S7. Boxplots showing the normalized number of IgA/IgG/IgM+IgD CDR3s in AML and non-tumor groups. The average expression value of 11 housekeeping genes (C1orf43, CHMP2A, EMC7, GPI, PSMB2, PSMB4, RAB7A, REEP5, SNRPD3, VCP, VPS29) was used to normalize the number of IgA, IgG, IgM, and IgD CDR3s in each sample. The p values were calculated using the two-sided Wilcoxon rank-sum test. * $p<0.05$, ** $p<0.01$, *** $p<0.001$, n.s. indicates not significant.

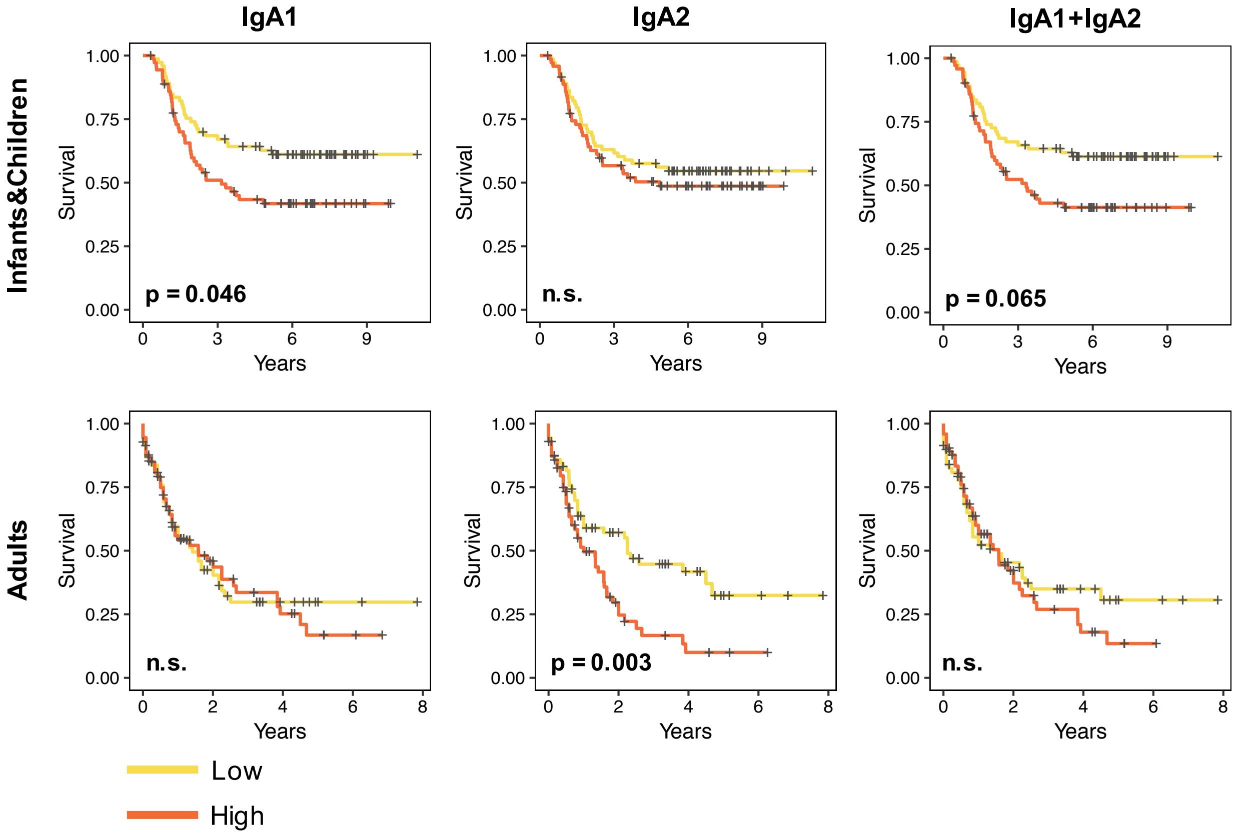

Fig. S8. Kaplan-Meier curves showing the survival difference relative to IgA fraction in pediatric and adult AML. Samples were divided into IgA1/IgA2/(IgA1+IgA2) ratio high (≥cutoff, orange) and IgA1/IgA2/(IgA1+IgA2) ratio low (<cutoff, yellow) group by the median fraction of this ratio in pediatric/adult AMLs. The statistical significances were estimated using Log-rank test.

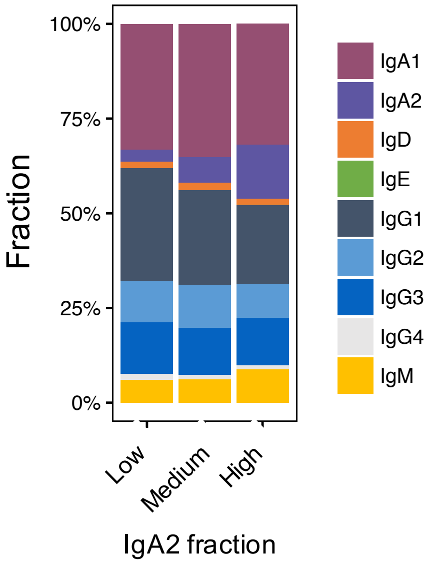

Fig. S9. Distribution of 9 Ig isotypes across adult AML IgA2 low (0-5%, n=64), medium (5%-10%, n=64) and high (>10%, n=23) group.

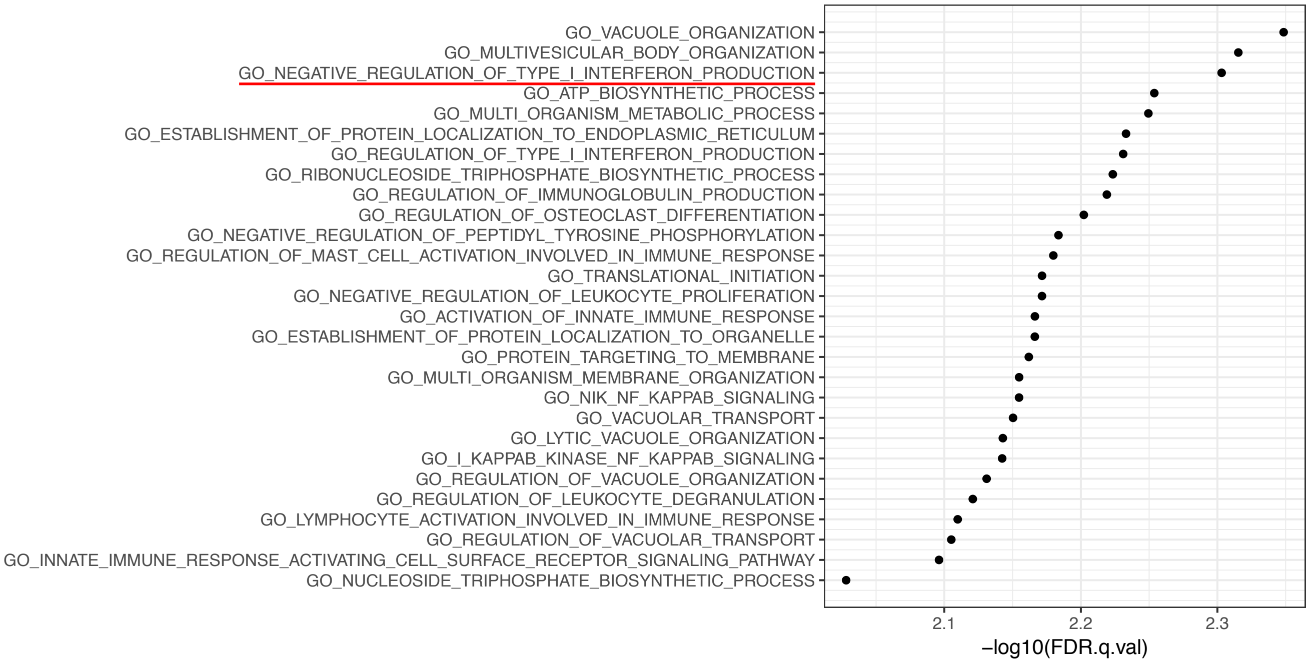

Fig. S10. The enriched GO terms with IgA2 fraction in adult AML. GSEA analysis was performed on a pre-ranked gene list to examine the enriched GO terms. The ranking of the gene list was based on the coefficient of Spearman's rank correlation with IgA2 fraction in adult AML.

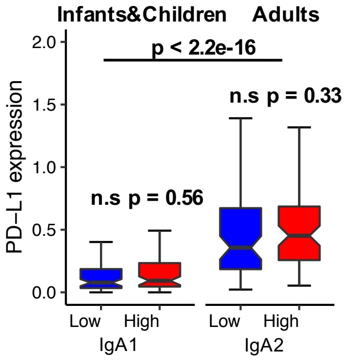

Fig. S11. Boxplot showing the expression level of PD-L1 in pediatric AML IgA1 high/low group and adult AML IgA2 high/low group. The p values were calculated using two-sided Wilcoxon rank-sum test.

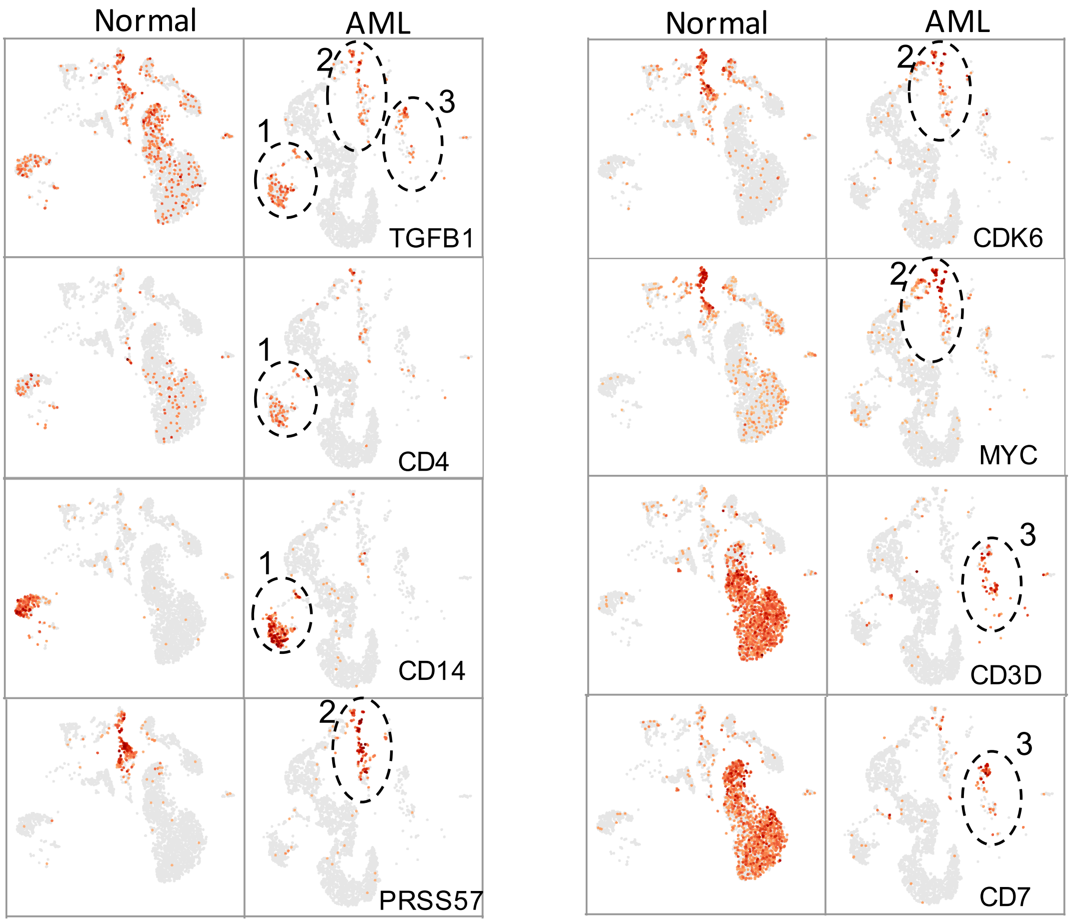

Fig. S12. t-SNE plots showing the expression level of different gene makers in normal and AML (M6, one patient) peripheral blood single cell RNA-seq data. Loupe Cell Browser was used to visualize the gene expression data. TGFB1 are highly expressed in three major cell clusters, including CD4+CD14+ monocytes, PRSS57+MYC+ neutrophils, and CD3+CD7+ T cells.
