# Supplementary material for: Immune receptor repertoires in pediatric and adult acute myeloid leukemia
Source: Genome Med. 2019 Nov 26;11:73. doi: 10.1186/s13073-019-0681-3 (PMC6880565; doi:10.1186/s13073-019-0681-3)
Supplement: Supplementary file 3 — Additional file 3. Supplementary methods. [file 13073_2019_681_MOESM3_ESM.pdf]

Supplementary Methods

Detection of TCR and BCR CDR3 sequences from AML and non-tumor samples

TRUST3.0.1 [1,2] (https://bitbucket.org/liulab/trust) was applied to all the RNA-seq samples using the following commands:

trust -f sample_name.bam -g hg38 -c -E for TCR detection;

trust -f sample_name.bam -g hg38 -c -B for IgH detection;

trust -f sample_name.bam -g hg38 -c -B -L for IgL, IgK detection.

Analysis of TCR and BCR CDR3 sequences

The clonotype diversity of T/B cells was estimated by TCR/BCR CDR3s per kilo TCR/BCR reads (CPK) in each sample. To investigate potential viral-related TCRs, we collected 4,872 (738 Cytomegalovirus, 921 Epstein-Barr virus, and 3,213 Flu) virus related β-CDR3s corresponding to eight common peptide-MHC complexes from previous research [3]. This enabled us to evaluate the number of virus-related TCR β-CDR3 divided by the total number of unique complete TCR β-CDR3s in each group (Fig. 1c). The virus-related TCR β-CDR3s were matched by both amino acid sequence and HLA information in each sample.

γδ T cell fraction was estimated by the total number of γ or δ-CDR3s divided by the total number of TCR CDR3s in each sample. For each pair of δ-CDR3s, we performed local sequence alignment with BLOSUM62, using gap opening penalty of 1 and gap extension penalty of 2. The similarity between two δ-CDR3s was evaluated by the identical positions in aligned sequences divided by the length of shorter sequence. Hierarchical clustering was performed on the δ-CDR3 similarity matrix in Fig. 2b. To obtain the conversion pattern of sequences in Cluster1 (Fig. 2c), ClustalW as implemented in the R package msa[4] was used for multiple local sequence alignment.

Other analysis

TPM (transcripts per million) was calculated based on the gene read count file to evaluate the gene expression level in each sample. GSEA [5] analysis was performed on a pre-ranked gene list to examine the enriched GO terms. The ranking

of the gene list was based on the coefficient of Spearman's rank correlation with IgA2 fraction in adult AML. Processed single cell gene expression data of one M6 AML patient [6] was downloaded from the official website of 10x genomics. Loupe Cell Browser was used to visualize the AML single cell RNA-seq data. OptiType [7] was used to identify the HLA class I typing of all the samples.

Statistical analysis

All the statistical tests and survival curves were implemented using R and the code was provided in https://bitbucket.org/jianz10/aml_immune_repertoire/.

References

1. Li B, Li T, Wang B, Dou R, Zhang J, Liu JS, et al. Ultrasensitive detection of TCR hypervariable-region sequences in solid-tissue RNA-seq data. Nat Genet. 2017;49:482–3.

2. Hu X, Zhang J, Liu JS, Li B, Liu XS. Evaluation of immune repertoire inference methods from RNA-seq data. Nat Biotechnol. 2018;36:1034.

3. Glanville J, Huang H, Nau A, Hatton O, Wagar LE, Rubelt F, et al. Identifying specificity groups in the T cell receptor repertoire. Nature. 2017;547:94–8.

4. Bodenhofer U, Bonatesta E, Horejš-Kainrath C, Hochreiter S. msa: an R package for multiple sequence alignment. Bioinformatics. 2015;31:3997–9.

5. Subramanian A, Tamayo P, Mootha VK, Mukherjee S, Ebert BL, Gillette MA, et al. Gene set enrichment analysis: a knowledge-based approach for interpreting genome-wide expression profiles. Proc Natl Acad Sci U S A. 2005;102:15545–50.

6. Zheng GXY, Terry JM, Belgrader P, Ryvkin P, Bent ZW, Wilson R, et al. Massively parallel digital transcriptional profiling of single cells. Nat Commun. 2017;8:14049.

7. Szolek A, Schubert B, Mohr C, Sturm M, Feldhahn M, Kohlbacher O. OptiType: precision HLA typing from next-generation sequencing data. Bioinformatics. 2014;30:3310–6.
